# Median-joining network analysis of SARS-CoV-2 genomes is neither phylogenetic nor evolutionary

**DOI:** 10.1073/pnas.2007062117

**Published:** 2020-05-07

**Authors:** Santiago J. Sánchez-Pacheco, Sungsik Kong, Paola Pulido-Santacruz, Robert W. Murphy, Laura Kubatko

**Affiliations:** ^a^Centre for Biodiversity, Royal Ontario Museum, Toronto, ON M5S 2C6, Canada;; ^b^Department of Evolution, Ecology, and Organismal Biology, The Ohio State University, Columbus, OH 43210;; ^c^Programa Ciencias de la Biodiversidad, Instituto de Investigación de Recursos Biológicos Alexander von Humboldt, Bogotá, 111311, Colombia;; ^d^Department of Ecology and Evolutionary Biology, University of Toronto, Toronto, ON M5S 3B2, Canada;; ^e^Department of Statistics, The Ohio State University, Columbus, OH 43210

Tracking the spread of pandemics and the evolution of the underlying pathogens are effective tools for managing deadly outbreaks. Forster et al. (1) use a median-joining (MJ) network (MJN) and its Steinerization process to investigate the evolution of severe acute respiratory syndrome coronavirus 2 (SARS-CoV-2), which causes coronavirus disease 2019 (COVID-19) within humans. They claim to “assist in tracing infection pathways and designing preventive strategies” by analyzing 160 genomes sampled worldwide. However, their assertation that “this method can contribute to an understanding of coronavirus evolution” is based on a misunderstanding of both the method and its interpretation. Because their work may have profound implications for understanding and managing the COVID-19 global pandemic, scrutiny is necessary.

MJNs are not an appropriate representation of viral evolution. Although Forster et al. (1) state their use in “enabling the visualization of a multitude of optimal trees,” MJNs actually portray only relatedness, rather than strict-sense phylogeny (2, 3). Even so, this interpretation is problematic in that such a network does not reflect the important biological features thought to underlie viral evolution, such as recombination and horizontal gene transfer, making MJNs inappropriate in this setting. Indeed, the cycles present in an MJN provide no information about the evolutionary history of the sequences due to the absence of direction (2). Forster et al.’s (1) misguided attempts to apply concepts and terms such as “phylogenetic network,” “ancestral,” and “phylogenetic clusters” in their interpretation ignore the fact that MJ demonstrably fails in these interpretations (2, 3). Even if MJNs did admit a strict phylogenetic interpretation, phylogenies do not directly trace transmission history (4, 5), though Forster et al. (1) devote much of their report to this inaccurate interpretation.

Additionally, MJNs are constructed using distance-based criteria, which is inappropriate for modeling the mutational process in viruses (6). In fact, the implication that MJNs reflect phylogenetic signal in the traditional sense has previously been challenged by Kong (3), who compared inference based on MJNs with Bayesian model-based phylogenetic inference for 85 published datasets and found substantial disagreement between inferred MJNs and posterior distributions on phylogenies, indicating that the two methods provide different measures of relatedness in a phylogenetic sense. Neither is the widespread use of the MJN method (1) sufficient justification for its (mis)application to the SARS-CoV-2 data.

The outgroup comparison by Forster et al. (1) is particularly problematic and leads to erroneous conclusions about the directionality of evolutionary changes. For example, their outgroup does not root at A, but rather A itself is derived from one of two possible ancestral viruses with this rooting. The MJ option used for rooting (user guide, https://www.fluxus-engineering.com/) merely links the “outgroup” sequence (i.e., nonconspecific; here, a bat coronavirus with substantial sequence divergence) to the most similar sequence of the already-produced “ingroup” network. Therefore, it neither roots the ingroup topology nor polarizes character transformations (2, 3).

The authors’ misinterpretation of MJNs fosters misconceptions, inaccuracies, and misrepresentations of fundamental phylogenetic principles. Thus, unfortunately, Forster et al.’s study (1) misleads more than illuminates an understanding of the evolutionary history of SARS-CoV-2 in humans.
